# Optimized Irrigation and Nitrogen Fertilization Enhance Sorghum Yield and Resilience in Drought-Prone Regions

**DOI:** 10.3390/plants14182913

**Published:** 2025-09-19

**Authors:** Binita Thapa, Ripendra Awal, Ali Fares, Anoop Valiya Veettil, Almoutaz Elhassan, Atikur Rahman

**Affiliations:** Cooperative Agricultural Research Center, College of Agriculture, Food, and Natural Resources, Prairie View A&M University, Prairie View, TX 77446, USA

**Keywords:** nitrogen rates, irrigation levels, crop growth, phenology, yield

## Abstract

Sorghum [*Sorghum bicolor* (L.) Moench] is a vital cereal crop in semi-arid regions, but its productivity is often limited by water scarcity and inefficient nitrogen (N) management. While previous studies have explored the individual effects of irrigation and N application, limited research has examined their combined influence on sorghum growth, phenology, and yield. This study aimed to assess the impacts of four irrigation levels (0, 75, 100, and 125% of irrigation as I_0_, I_75_, I_100_, and I_125_) and four N application rates (0, 90, 180, and 360 kg N ha^−1^ as N_0_, N_90_, N_180_, and N_360_) on sorghum performance to identify optimal water and nutrient management strategies. A field experiment was conducted over two growing seasons, measuring plant height, number of leaves, panicle length, flowering percentage, visual maturity rating, biomass, and grain yield using a split-plot design, with irrigation as the main factor and N rates as subplots with three replications. Key findings revealed that irrigation and N application significantly influenced plant height, number of leaves, panicle length, biomass, and grain yield. The highest plant height (58.06 cm), number of leaves (10.25), and panicle length (26.60 cm) were observed under I_125_. Compared to the I_0_ treatment, the increase in visual maturity rating in I_75_ and I_100_ was 64.91% and 249.70%, respectively. The highest biomass (3.25 t/ha) was observed for treatment I_125_, while the highest grain yield (2.40 t/ha) was for I_100_. Compared to N_0_, treatments N_90_, N_180_, and N_360_ increased grain yield by 64.8, 66.4, and 81.6%, respectively. The Pearson correlation analysis revealed significant positive relationships among key crop growth, phenology, and yield traits. The maturity rating correlated highest with flowering percentage (0.76). The response to N application was more pronounced under well-watered conditions, with the steepest grain yield increase observed under N_360_. In water-limited conditions, higher N rates did not improve yield. Based on these results, applying irrigation at 100% of the crop water requirement and at least 180 kg N ha^−1^ is recommended to enhance sorghum resilience, resource-use efficiency, and sustainable production, particularly in water-scarce regions.

## 1. Introduction

The main challenges faced in arid and semi-arid areas are escalating drought conditions, erratic rainfall patterns, and decreasing availability of irrigation water [1], which pose a significant threat to global food security. The UN’s Intergovernmental Panel on Climate Change (IPCC) and other international reports show that climate and extreme weather events are already affecting crop yields and increasing global food insecurity [2,3,4]. As the world’s population grows, the projected increase in food demand will place further strain on already limited water resources, a challenge underscored by the fact that nearly four billion people globally already experience water scarcity [5]. This makes the development of sustainable agricultural practices that enhance soil and crop water use efficiency a critical global priority [6,7,8,9]. In the United States, climate change is intensifying water risks, with recent reports highlighting the growing threats of both water scarcity and excess to U.S. agriculture, particularly in the western and midwestern states [10,11,12]. Due to its vulnerability to drought, Texas, the nation’s second-largest sorghum producer [13], faces significant challenges in maintaining crop yields. Farmers and researchers are exploring new ways to sustain crop production and boost water efficiency while using less water to combat this. This is crucial for ensuring the long-term viability of agriculture in the state’s semi-arid regions.

Grain sorghum [*Sorghum bicolor* (L.) Moench] possesses substantial potential for broader utilization, including food, feed, fuel, construction material, and various industrial applications [14,15]. Due to the high-water use efficiency and resilience to drought and heat, sorghum could be a viable substitute for maize in water-scarce environments [16]. However, even this resilient crop is not immune to the adverse effects of water and nutrient limitations.

Farmers can directly impact crop productivity by managing two vital elements: water availability and nitrogen nutrition. Thus, irrigation and fertilizers are principal inputs of agricultural production, which substantially influence crops’ quality and quantity [17]. Reduced water availability disrupts water and nutrient uptake, reducing chlorophyll levels and compromising crop growth and yield [18,19]. Sorghum has demonstrated resilience in adverse climatic conditions, such as drought and temperature fluctuations, and exhibits robust growth even in marginal soil environments [20,21]. Sorghum can access water from soil depths as deep as 270 cm [22]. Sorghum has been recognized as a water-efficient crop that efficiently converts the available water into dry matter compared to other crops [23]. Although sorghum is resilient to prolonged water stress [24], water stress conditions adversely affect plant height, chlorophyll content, dry matter production, and yield [25,26,27,28]. The study showed that water stress conditions during vegetative and reproductive stages could reduce sorghum yield by 36% and 55%, respectively [29]. Jahansouz et al. [30] also reported that a 25% reduction in irrigation water resulted in a decrease of 13% in the dry matter yield of sorghum.

Nitrogen (N) is a key nutrient and often the most limiting factor in modern crop production [31,32]. It is essential for synthesizing amino acids, proteins, and pigments [33]. Studies have shown that increasing N rates improves sorghum’s growth, development, and yield [34]. Nonetheless, a substantial portion of N fertilizer, ranging from 50% to 70%, is lost to the environment due to leaching, volatilization, runoff, and nitrous oxide emissions, posing a threat to environmental pollution [35]. Excessive irrigation and N fertilization can lead to nitrate leaching, posing a water table contamination risk [36]. Additionally, N fertilizer impacts soil health through acidification. Moreover, over recent years, there has been a rise in fertilizer prices and an increased awareness of water conservation practices. Hence, adequate soil moisture is crucial for efficient N utilization [37], as water-deficient soils limit nitrogen availability and reduce crop yields. Enhancing nitrogen management within irrigated production systems is crucial to maximize profits and minimize adverse environmental impacts [38].

Research shows that while irrigation and nitrogen affect sorghum, their combined impact on yield varies by location. For example, studies in China and Turkey demonstrate that optimizing the interaction between these two factors is essential for maximizing crop output [39,40]. Research from the southeastern U.S. emphasizes that supplemental irrigation and nitrogen are crucial for reducing the effects of water stress in humid climates [41]. While extensive research has explored the individual effects of water stress and nitrogen deficiency on sorghum, a significant gap remains in understanding their interactive effects on crop growth, physiology, and water use efficiency, particularly within specific regional contexts like Texas. Developing an integrated management strategy that optimizes irrigation and nitrogen is crucial for sustainable sorghum production, as the complex interaction between these two factors requires strong support from research-based information to maximize profitability and minimize environmental impacts in water-stressed agricultural systems. Therefore, this study was conducted in Southeast Texas to evaluate the combined effects of different irrigation levels and nitrogen rates on sorghum’s growth, phenology, and yield. We hypothesized that both individually and interactively, water stress and nitrogen limitation would negatively impact these key crop parameters.

## 2. Materials and Methods

### 2.1. Study Site and Treatments

The field experiment was conducted at the College of Agriculture, Food, and Natural Resources farm (Latitude: 30°5′33.95″ N, Longitude: 95°58′43.14″ W), Prairie View A&M University, Prairie View, Texas, during the 2023 and 2024 sorghum growing season. The mean annual rainfall is 1195 mm based on monthly rainfall data from 1991 to 2024 (PRISM data: http://www.prism.oregonstate.edu/, accessed on 11 August 2025), with mean minimum and maximum temperatures of 14.2 and 26.0 °C, respectively. The experimental site’s primary soil type is Wockley fine sandy loam. According to the U.S. Soil Taxonomy, the taxonomic classification of the Wockley Series is as follows: fine-loamy, siliceous, semiactive, hyperthermic Plinthaquic Paleudalfs [7].

**Figure 1:**
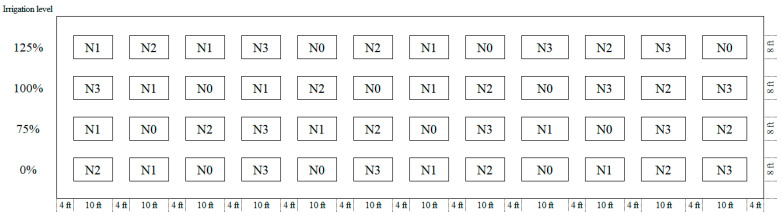
Field layout of the experimental design at Prairie View A&M University. The plot design includes irrigation levels and nitrogen rates arranged in four replications. N1, N2, N3, and N4 represent no nitrogen, half the recommended rate, the recommended rate, and double the recommended N rate, respectively. The 100% irrigation level is a fully irrigated treatment with no water stress.

**Figure 2:**
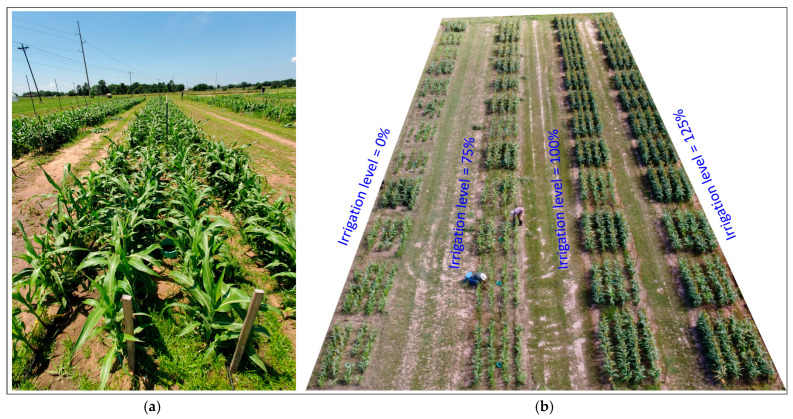
(**a**) Plots of 100% irrigation level (9 June 2023) and (**b**) drone imagery of experimental field (30 June 2023).

The study was conducted in a split-plot design with four irrigation levels (supply 0, 75, 100, and 125% of irrigation as I_0_, I_75_, I_100_, and I_125_) as the main factor and four nitrogen rates (0, 90, 180 and 360 kg N ha^−1^ as N_0_, N_90_, N_180_, and N_360_) as the sub-factor randomized with three replications during the 2023 and 2024 growing seasons in Prairie View, Texas, USA (Figure 1). The 100% irrigation level is a fully irrigated treatment with no water stress. Figure 2 illustrates the meteorological conditions observed during the 2023 and 2024 growing seasons.

### 2.2. Land Preparation and Planting Operations

Each subplot consisted of four planting lines, 3 × 2.5 m long, with 2 m paths separating them within the plot. The soil samples were analyzed before applying nitrogen treatments. The total nitrogen required during the growing season was estimated as 180 kg N ha^−1^.

The soil analysis results showed phosphorus (P) and potassium (K) content as 86 and 84 mg kg^−1^ of soil, respectively, so P and K were not applied during the growing season. N_90_, N_180_, and N_360_ plots’ nitrogen was supplied through urea fertilizer. The urea was applied during sowing, and it was mixed well. The sowing operation was conducted on 20 April and 26 April for 2023 and 2024, respectively. Sorghum seeds were seeded 30 cm apart within rows and 60 cm between rows. In this study, a drip tape irrigation approach was applied (with a diameter of 16 mm and a drip distance of 10 cm). IrrigWise, a web-based irrigation scheduling tool, was utilized to determine irrigation requirements by incorporating near-real-time site-specific data on rainfall, reference evapotranspiration, soil hydrologic properties, and plant growth stages [42].

### 2.3. Data Collection and Measurement

Plant height (height from the base of the plant to the base of the fully emerged top leaf), number of leaves, and panicle length (length of the panicle from the node where the first panicle branch emerges to the tip of the panicle) were measured as crop growth parameters. Days to 50% heading (days from planting to emergence of 50% of the flowers from each plot) and visual maturity rating (redness on grain color of panicle from each plot) after 45 and 75 days of planting, respectively, were measured as crop phenology traits. Biomass (plant weight after harvesting and drying) and grain yield (grain weight after the panicles were threshed, cleaned, and dried) were measured as yield traits.

### 2.4. Statistical Analysis

Analysis of variance (ANOVA) was performed using Statistix 10 software (Analytical Software 2015, Tallahassee, FL, USA) to test the effects of irrigation levels and nitrogen rates on plant growth parameters (plant height, number of leaves, and panicle length), crop phenology (flowering percentage and visual maturity rating), and yield (biomass and grain yield) traits. The least significant difference (LSD) was used for mean separations of treatment effects when the F-test was significant at *p*-value < 0.05. R-software (RStudio 2020, Boston, MA, USA) prepared the bar graphs, response graphs, and correlation matrix.

## 3. Results and Discussion

### 3.1. Crop Growth Parameters (Plant Height, Number of Leaves, and Panicle Length) Traits

**Figure 3:**
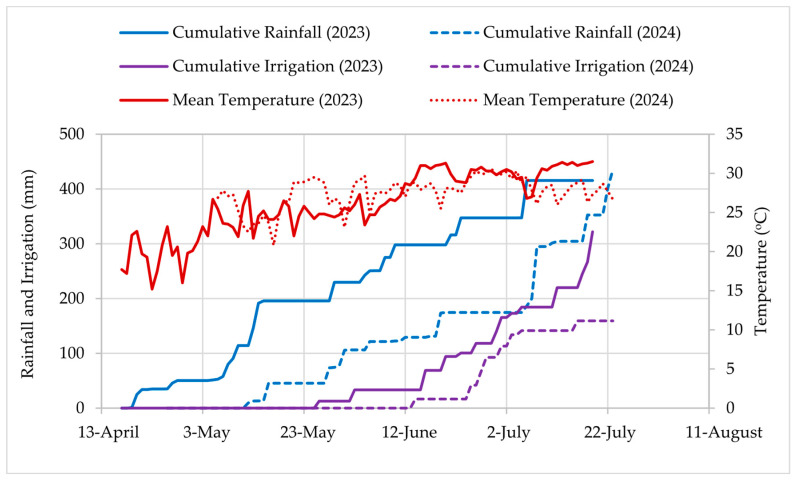
Cumulative rainfall, irrigation (100% irrigation level), and mean temperature during the experimental site’s 2023 and 2024 growing seasons.

The results showed that irrigation and nitrogen significantly affected plant height, number of leaves, and panicle length (Table 1). The highest plant height and number of leaves were obtained in the I_125_ treatment (Table 2). No significant differences in the plant height and number of leaves were observed for N_90_, N_180_, and N_360_, whereas the minimum was obtained in N_0_. Compared to the I_0_ treatment, the increase in panicle length in I_75_, I_100_, and I_125_ was 5.54, 14.16, and 18.02%, respectively. The number of leaves and panicle length were significantly greater in the 2024 growing season than in 2023. Compared to the growing season in 2023, the panicle length increased by 4.31% in 2024. The plant growth rate is strongly influenced by environmental factors such as light, temperature, CO_2_, humidity, and wind [43]. The favorable weather conditions in 2024 might have resulted in greater crop growth parameters than in 2023. In 2023, cumulative rainfall increased rapidly from early May; in contrast, 2024 experienced minimal rainfall early in the season (Figure 3). Similarly, for N_180_, the plant height and panicle length increased by 10.96% and 13.25% compared to N_0_, respectively. In this experiment, water stress conditions reduced plant growth parameters: plant height, number of leaves, and panicle length, which could be attributed to insufficient moisture in the soil profile [44,45]. However, I_75_, I_100_, and I_125_ did not change these parameters. Water stress conditions reduce the chlorophyll content [46], leading to the breakdown of existing chlorophyll in the leaves and inhibiting the synthesis of new chlorophyll [47]. Similar results were obtained for the N rate. Consistent with our result, Heitman et al. [31] observed an increased plant height with an increased N rate. At the same time, Cao et al. [48] observed a reduction in sorghum plant growth and an increased ammonium toxicity due to increased N rate. The observed decrease in plant height, number of leaves, and panicle length under water-stressed conditions demonstrates the phenotypic plasticity of sorghum, a crucial adaptive mechanism for survival in challenging environments. This is consistent with findings in other crops, such as spineless safflower, where morphological traits were also found to be highly responsive to environmental stress [49]. While establishing a clear link between water and nitrogen levels and sorghum growth, our study suggests that future research should also consider how these factors interact with elevated CO_2_ and heat stress to better predict crop performance under climate change [50,51].

### 3.2. Crop Phenology (Flowering Percentage and Visual Maturity Rating) Traits

The results showed that irrigation and nitrogen significantly affected heading percentage (Table 1). Irrigation levels I_100_ and I_125_ did not significantly differ in either crop phenology. The lowest maturity rating was observed for the I_0_ treatment, while no significant differences were observed for the irrigation treatment. Compared to the I_0_ treatment, the increase in visual maturity rating in I_75_ and I_100_ was 64.91% and 249.70%, respectively (Table 2). The number of flower heads was significantly greater in the growing season of 2024 compared to 2023. Compared to the growing season in 2023, the flower head increased by 44.27% in 2024. Like our experiment, Ndlovu et al. [52] observed that drought stress significantly delays floral initiation and panicle development. Water-stressed conditions can delay the flowering from 73 to 90 days after sowing relative to well-watered crops [53]. Some studies have reported that water stress can reduce photosynthate production, which may cause a halt or delay in flower development, as water stress inhibits the supply of photosynthates [54]. Our study demonstrates that sorghum phenology is highly sensitive to water and nitrogen stress, a key factor in forecasting crop performance under future climate scenarios with increasing drought frequency. This aligns with challenges reported in other regions, such as the documented effects of climate change on alfalfa yields in Iran [50].

### 3.3. Yield (Biomass and Grain Yield) Traits

**Figure 4:**
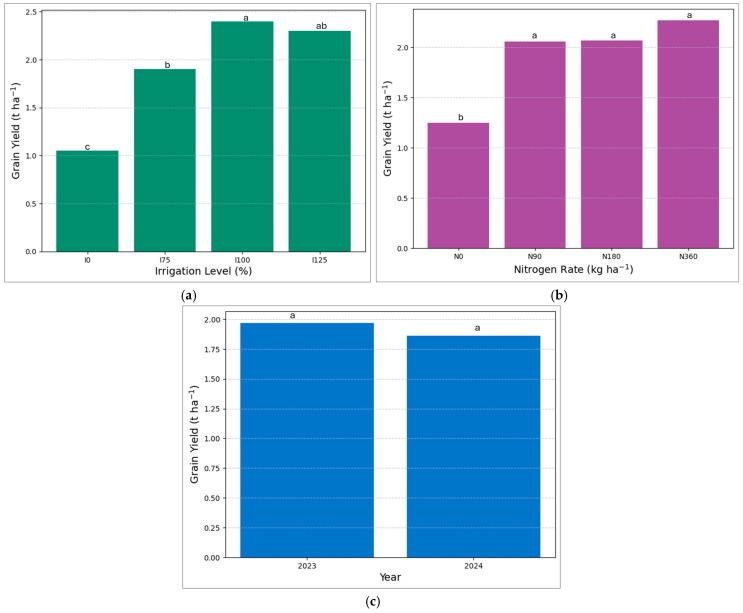
Mean comparisons of effects of irrigation levels (**a**), nitrogen rates (**b**), and year (**c**) for sorghum grain yield. The same letters do not have statistically significant differences at a 5% level of probability according to LSD.

The results showed that irrigation and nitrogen significantly affected biomass and grain yield (Table 1). No significant difference was observed in grain yield for 2023 and 2024 (Figure 4c). Although the timing of rainfall and irrigation differed between the two years, the total water availability (rainfall plus irrigation) by late July (Figure 3) was comparable, which, along with similar temperature trends, likely explains the lack of significant yield differences between the two seasons. The highest biomass was observed for treatment I_125_, while the highest grain yield was for I_100_ (Table 2, Figure 4a). The lowest biomass and grain yield were obtained for treatment N_0_. The treatments I_100_ and I_125_ were not significantly different for biomass. Compared to treatment I_100_, treatment I_75_ and I_0_ reduced biomass by 25.23% and 34.89%, respectively. Our experiment aligns with Anjum et al. [55], who observed an increase in sorghum yield with an increase in irrigation level. Similarly, Farhadi et al. [56] found that the biomass yield was decreased due to limited irrigation. Water stress conditions significantly affect crop yield by disrupting growth and development due to various inhibitory effects on biochemical and physiological processes [55]. Drought stress decreases plant photosynthesis by limiting nutrient uptake and assimilation, reducing growth and biomass accumulation [57,58]. Scordia et al. [59] observed that reducing irrigation rates by 25% and 36% throughout the season led to biomass yield decreases of 17% and 36%, respectively, compared to full irrigation due to lower leaf area index and reduced radiation use efficiency.

Drought stress significantly reduces grain yields by decreasing seed size, seed number, grain weight, and other agronomic traits [60,61]. It has also been found that drought stress during the vegetative and reproductive stages reduced grain yield by over 36% and 55%, respectively [29]. Kapanigowda et al. [62] revealed that pre-flowering and post-flowering drought stress significantly reduce grain yield. Compared to N_0_, the results showed that treatments N_90_, N_180_, and N_360_ increased biomass by 38.81, 47.26, and 58.21%, respectively (Table 2). Similarly, compared to N_0_, treatments N_90_, N_180_, and N_360_ increased grain yield by 64.8, 66.4, and 81.6%, respectively (Figure 4b). Similarly to our experiment, previous research has shown that increased N rate increased biomass and yield [63,64]. Reduced nitrogen fertilizer application may lead to decreased biomass yield and water use efficiency, likely due to diminished activity of photosynthetic enzymes [64,65]. Irrigation enhances fertilizer efficiency, making soil fertilization more cost-effective under optimal water conditions than under water-scarce conditions due to the positive relationship between irrigation and fertilization [66,67]. Other researchers also observed that combining the fertilizer with irrigation increases crop yield [68,69,70]. Although I_125_ produced the highest biomass (3.25 t/ha), grain yield (2.30 t/ha) did not exceed that of I_100_ (2.40 t/ha), resulting in a slightly lower harvest index (HI) of 0.738 vs. 0.717, indicating that excessive irrigation favored vegetative growth over reproductive sink development. Similarly, a nitrogen rate of 90 kg ha^−1^ (HI = 0.738) efficiently converted biomass to grain, whereas higher N rates of 180 and 360 kg ha^−1^ (HI = 0.699 and 0.714, respectively) mainly increased vegetative growth, underscoring the need for optimized irrigation and nitrogen management to achieve balanced source–sink allocation. Nitrogen levels affect grain yield at a molecular level by mediating the expression of specific genes, ultimately influencing biomass and crop output [71]. Beyond the physiological and yield-based results, the financial implications are also paramount for farmers. Optimizing specific irrigation and nitrogen inputs is crucial for achieving economic efficiency using nitrogen fertilizer [72]. In this experiment, the higher irrigation and nitrogen did not significantly improve the yield; instead, they led to the wastage of resources.

**Figure 5:**
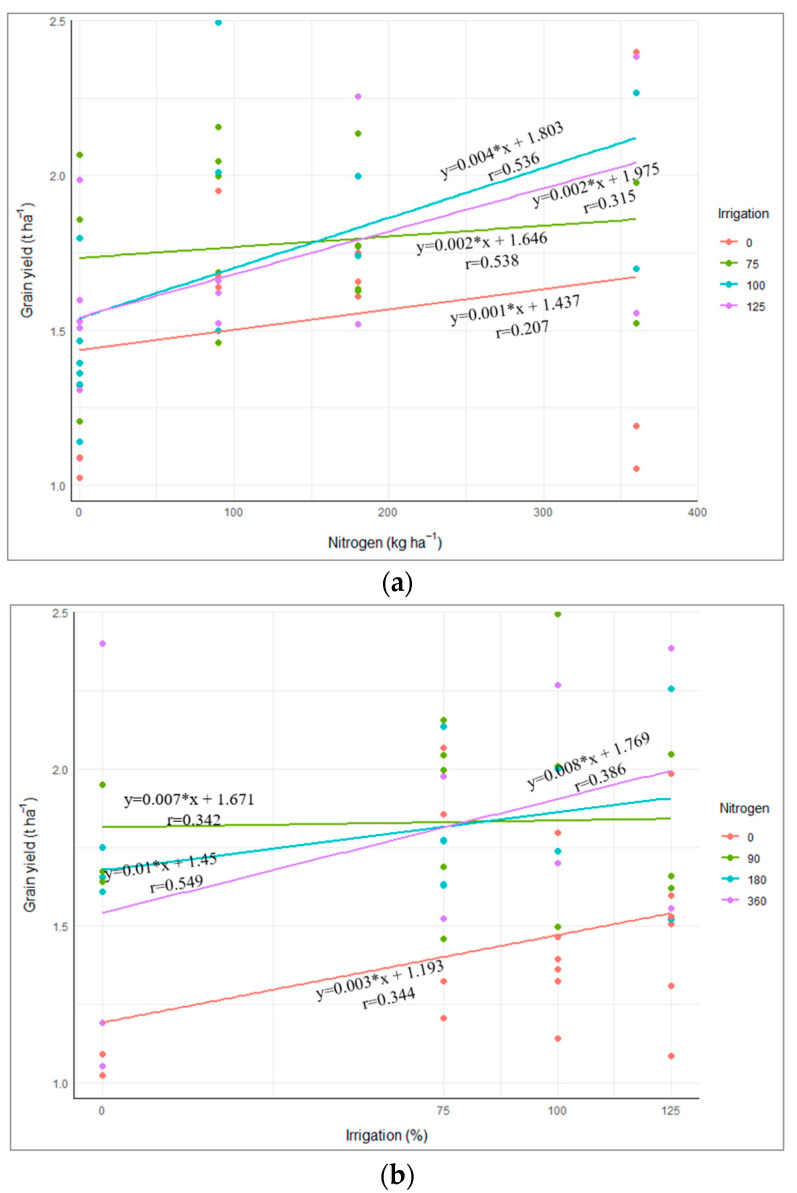
The grain yield response to the nitrogen rate under different irrigation levels (**a**) and to the irrigation levels under different nitrogen rates (**b**). Irrigation 0, 75, 100, and 125 represent irrigation levels: the supply of 0, 75, 100, and 125% of the fully irrigated treatment, respectively; Nitrogen 0, 90, 180, and 360 represent nitrogen rates: application of 0, 90, 180, and 360 kg N ha^−1^, respectively.

The regression analysis of grain yield response to N application under varying irrigation levels (I_0_, I_75_, I_100_, and I_125_) demonstrated distinct trends (Figure 5a). The grain yield in the I_100_ and I_125_ exhibited an increasing trend with rising nitrogen levels. In contrast, I_75_ did not show an increasing trend of grain yield with increasing nitrogen application. Like this experiment, Flynn et al. [73] reported that lower nitrogen rates were sufficient to optimize yield under limited water conditions compared to full irrigation. Water deficit conditions reduce crop yield by impairing fertilizer use efficiency [74,75]. The sorghum yield response to the nitrogen application in I_75_ plots suggests a hormetic effect [76], where a beneficial increase in nitrogen becomes detrimental beyond a certain threshold. Excess nitrogen in maize leaves can trigger stomatal closure, reduce stomatal conductance, negatively impact photosynthesis, and make plants more vulnerable to drought stress [77]. Therefore, optimizing irrigation enhances fertilizer efficiency and soil nitrogen availability, promoting nitrogen uptake and translocation to the grain, ultimately leading to higher crop yields [78]. Furthermore, the highest grain yield was observed under I_100_ with an increasing N application. The lower r values for higher irrigation levels suggest that additional factors, such as soil properties and nutrient interactions, may influence yield response under well-watered conditions.

The regression analysis of grain yield response to irrigation levels under varying nitrogen levels (N_0_, N_90_, N_180_, and N_360_) demonstrated distinct trends (Figure 5b). At the nitrogen level N_360_, the grain yield showed the steepest positive response to increasing irrigation (r = 0.386). Similarly, under N_180_, grain yield increased with irrigation, but with a lower slope than under N_360_. In contrast, at N_0_, the response to irrigation was minimal, highlighting the impact of limited irrigation on yield when nitrogen is deficient. These results highlight that optimizing nitrogen and water inputs is crucial for maximizing grain yield, particularly under water-limited conditions.

The results highlight the potential for integrating optimized irrigation and nitrogen management into climate-smart cropping systems. Despite variations in rainfall timing, the consistent yield across years suggests that well-timed irrigation can enhance drought resilience and reduce the risk of yield loss under variable weather conditions. This study provides practical guidance for farmers by showing that irrigation at 100% of crop water requirement, combined with at least 180 kg N ha^−1^, achieves near-maximum sorghum yield while reducing unnecessary input costs. Understanding the interaction between water and nitrogen allows farmers to make informed management decisions and mitigate risks under drought or water-limited conditions. Additionally, the limited benefits from excessive nitrogen or irrigation emphasize the importance of precision management strategies that match crop requirements with resource availability. Adopting such practices can improve water and nutrient use efficiency, minimize environmental impacts, and support sustainable production in water-limited regions.

### 3.4. Correlation Matrix for Plant Growth, Phenology, and Yield Traits

**Figure 6:**
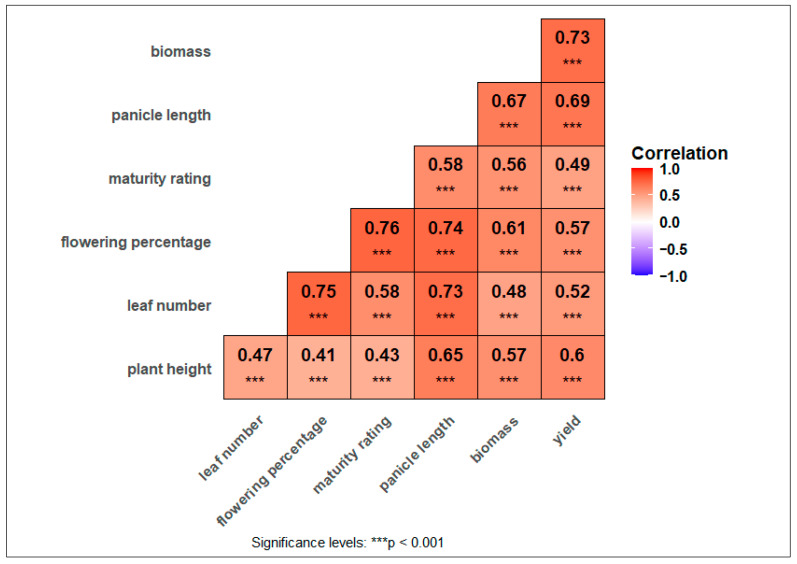
Correlation Heatmap showing relationships among plant height, leaf number, panicle length, maturity rating, flowering percentage, biomass, and yield in sorghum. Negative (blue color) or positive (red color) correlations between traits are identified by color (−1.00 to +1.00).

The Pearson correlation analysis revealed significant positive relationships among key crop growth, phenology, and yield parameters (Figure 6). Biomass exhibited a strong correlation with yield (r = 0.73). Similarly, plant height showed significant positive correlations with biomass (r = 0.57) and yield (r = 0.60), suggesting that taller plants contribute to greater biomass accumulation and yield potential. Thapa et al. [79] observed a positive correlation between plant height and biomass. Similarly, Vinoth et al. [80] observed that grain yield was positively correlated with plant height, leaf length, panicle length, and panicle weight. They also observed the positive association of days to flower with days to maturity, indicating that days to flowering are shorter, resulting in early maturity. Panicle length was positively correlated with biomass (r = 0.67), yield (r = 0.69), and plant height (r = 0.65). Deshmukh et al. [81] also observed a positive correlation of grain yield with panicle parameters, while Tiliye et al. [82] observed that days to flowering were positively correlated with maturity days and plant height but were negatively correlated with chlorophyll content, panicle length, and grain yield. Leaf number also significantly correlated with biomass, yield, and panicle length, reinforcing leaf number and providing more surface area for photosynthesis, photosynthates, and yield [83]. Maturity rating and flowering percentage were strongly associated with multiple parameters, particularly yield and biomass, indicating that timely maturity and efficient reproductive development are crucial factors in sorghum productivity. Notably, maturity rating had the highest correlation with flowering percentage (r = 0.76), further highlighting the synchrony between these two traits. Water deficit and nitrogen limitation significantly reduced flowering head percentages (I_0_: 17.1%, N_0_: 29.6%) and visual maturity ratings (I_0_: 22.1%, N_0_: 53.6%) (Table 2), reflecting prolonged time to anthesis and physiological maturity. These delays likely shortened the grain-filling period and disrupted source–sink balance, reducing yield under stressed conditions. These results align with other research where sufficient nitrogen and moisture availability enhance photosynthetic capacity during grain filling and grain development [84,85,86]. These findings underscore the importance of selecting agronomic management strategies for these traits to enhance sorghum productivity under varying environmental management conditions.

## 4. Conclusions

This study underscores the critical role of irrigation and nitrogen application in optimizing sorghum growth and productivity. Higher irrigation levels (I_100_ and I_125_) significantly improved plant height, number of leaves, and grain yield, with I_100_ achieving the highest yield of 2.30 t ha^−1^, representing a 119% increase compared to control irrigation. Similarly, higher nitrogen rates (N_90_, N_180_, and N_360_) significantly enhanced plant growth, phenology, and yield traits, with N_180_ producing a 66% greater yield than the control. Increased nitrogen application positively influenced growth parameters, but its effectiveness was more pronounced under adequate irrigation. Regression analysis demonstrated that grain yield response varied across irrigation and nitrogen levels, with a stronger correlation observed at higher nitrogen rates under well-watered conditions. The strong positive correlations among biomass, yield, plant height, and panicle length further emphasize the interdependence of growth traits in sorghum productivity. Water stress negatively impacted plant performance, reinforcing the need for balanced irrigation and nitrogen management. These findings provide valuable insights for developing sustainable environmental management strategies to improve sorghum production, particularly in semi-arid and water-limited regions. Applying 100% crop water requirement with moderate nitrogen (N_180_) maximizes yield and minimizes input risk, providing a practical strategy for sorghum farmers in semi-arid regions. Future research should explore the long-term impacts of combined water and nutrient management under varying climatic conditions and validate these optimal water–nitrogen combinations across a wider range of sorghum genotypes.

## Figures and Tables

**Table 1:** *p*-values obtained from ANOVA (Analysis of Variance) for the effect of year, irrigation, nitrogen, and their interactions with plant height, number of leaves, flowering percentage, panicle length, maturity rating, biomass, and yield.

SOV	df	Plant Height	Number of Leaves	Flowering Percentage	Panicle Length	Maturity Rating	Biomass	Yield
Year (Y)	1	0.2191	0.0000	0.0000	0.0008	0.4306	0.0079	0.3411
Irrigation (I)	3	0.0000	0.0000	0.0000	0.0000	0.0000	0.0000	0.0000
Nitrogen (N)	3	0.0013	0.0000	0.0000	0.0000	0.7807	0.0000	0.0000
Y × I	3	0.0031	0.0053	0.0000	0.4611	0.0000	0.0000	0.0000
Y × N	3	0.3185	0.2863	0.0992	0.0066	0.1577	0.0019	0.0544
I × N	9	0.6227	0.6271	0.3637	0.4716	0.3048	0.0000	0.2665
Y × I × N	9	0.7302	0.0654	0.0056	0.0979	0.9926	0.0181	0.1418

**Table 2:** The main effects of the year, irrigation, and nitrogen for plant height, leaf number, flowering, panicle length, maturity rating, and biomass.

Factors		Plant Height (cm)	Number of Leaves	Panicle Length (cm)	Flowering Head (%)	Visual Maturity Rating (%)	Biomass (t/ha)
Year	2023	55.54 a	8.98 b	24.14 b	38.09 b	53.46 a	2.87 a
	2024	53.94 a	10.40 a	25.18 a	54.95 a	55.35 a	2.60 b
Irrigation Level	I_0_	47.611 b	8.54 c	22.54 c	17.10 b	22.13 c	2.09 b
	I_75_	55.88 a	9.33 b	23.79 b	14.77 b	36.54 b	2.40 b
	I_100_	57.40 a	10.63 b	25.73 a	78.15 a	77.29 a	3.21 a
	I_125_	58.06 a	10.25 a	26.60 a	76.05 a	81.67 a	3.25 a
Nitrogen Level	N_0_	50.20 b	8.96 b	22.34 b	29.56 b	53.63 a	2.01 c
	N_90_	55.97 a	9.96 a	25.06 a	53.56 a	55.17 a	2.79 b
	N_180_	55.50 a	9.96 a	25.30 a	55.42 a	52.83 a	2.96 ab
	N_360_	57.29 a	9.88 a	25.97 a	47.53 a	56.00 a	3.18 a

The same letter indicates no significant difference among means by least significant difference (LSD) comparison of means at *α* = 0.05.

## Data Availability

The data presented in this study will be made available by the authors upon request.
